# Vom „digital divide“ zum „cyber divide“: Akzeptanz des digitalen Wandels – ein Vergleich zwischen der Wahrnehmung von Chancen und Risiken der Cyberwelt in der Onlinebevölkerung der Schweiz und der USA

**DOI:** 10.1365/s40702-023-00971-3

**Published:** 2023-05-10

**Authors:** Mascha Kurpicz-Briki, Christoph Glauser, Loris Schmid

**Affiliations:** 1 Applied Machine Intelligence Research Group, Berner Fachhochschule, Biel/Bienne, Schweiz; 2 Institut für angewandte Argumentenforschung, Genossenschaft IFAA, Bern, Schweiz

**Keywords:** Online-Suche, Suchverhalten, Online Wirkung Nutzerverhalten, Risiko-Affinität Schweiz/USA, Cyber-Chancen, Cyber-Risiken, Cyber divide, Digital divide, Online search, Search behavior, Digital impact, User behavior, Risk affinity Switzerland/USA, Cyber opportunities, Cyber risks, Cyber divide, Digital divide

## Abstract

Schweizer Nutzer*innen suchen dreimal häufiger nach Cyberrisiken als die User in den USA. Wir lebten aufgrund der Pandemie plötzlich in einer weitgehend digitalen Schweiz und 8,01 Mio. aktive Menschen nutzten in der Schweiz gemäss ITU die zahllosen Chancen des Internets. Ein Leben ohne Internet ist kaum mehr vorstellbar. Doch welches sind die wichtigsten Chancen und Risiken, welche die Bevölkerung tatsächlich beschäftigen? Wie verbreitet sind beispielsweise die Gefahren der Cyberwelt, wie etwa Hate Speech, Cyberbullying oder Cybergrooming? Welche Kanäle werden verwendet, um Informationen zu diesen Themen zu suchen und zu finden? Haben wir in der Schweiz im Vergleich zu den USA ein zu starkes Risikobewusstsein (Bias) gegenüber den Cyberthemen? Fokussieren sich die Schweizer*innen viel zu wenig auf die zahlreichen Online-Gelegenheiten und -Möglichkeiten währenddem die Amerikaner*innen dieses Potenzial voll ausnutzen? Dieses Thema untersucht die vorliegende Studie. Bis ins Jahr 2000 machte man sich Sorgen um den gleichberechtigten Zugang zum Internet und identifizierte einen *digital divide*. Heute ist dieses Thema, zumindest in den Industrieländern, vom Tisch. Man müsste heute vielmehr davon sprechen, dass manche Menschen grosse Angst haben vor den neuen Technologien und andere ganz selbstverständlich diese Chancen nutzen. Die Autor*innen identifizieren in dieser Studie einen neuen *cyber divide*, der heute primär zwischen Risiko-und Chancen-affinen Usern unterscheidet.

## Abstract (en)

Swiss users search for cyber risks three times more often than users in the USA. Due to the pandemic, we suddenly lived in a largely digital society, and according to the ITU, 8.01 mio. active people in Switzerland use the countless opportunities offered by the Internet on a regular basis. It is hard to imagine life without the Internet. But what are the most important opportunities and risks that actually concern the Swiss and the US population? For example, how widespread are the dangers of the cyber world, such as hate speech, cyberbullying or cybergrooming? What channels are used to search for and to find information about such topics? Do we have a much too strong risk awareness (bias) towards the risky cyber topics in Switzerland compared to the USA? Do Swiss users focus too little on the numerous online opportunities of the digital world, while Americans take full advantage of this potential without any concerns? This is what the present study is investigating. Until about the year 2000, people were concerned about equal access to the Internet and identified a “*digital divide”*. Today, this issue is no longer discussed, at least not in industrialized countries. The authors now rather speak of a new divide where people are afraid of the new technologies whereas others are fully profiting of the advantage of the online and digital opportunities as a matter of fact. Therefore, the authors identify a new “*cyber divide”* that today primarily divides between risk- and opportunity-aware users.

## Einführung

Während herkömmliche Umfragen den Nachteil haben, dass immer nur ein bestimmter Prozentsatz der Bevölkerung mitmacht und aus verschiedenen Gründen die Teilnehmenden nicht immer ehrlich antworten, basiert die vorliegende Studie auf den Vollerhebungsdaten zu den Suchvolumina der gängigsten Suchmaschinen, Social Media und e‑Shops unter Beizug von digitalen impact KPIs^1^. Untersucht wird, welche spezifischen positiven und negativen Wörter der Cyberwelten auf welchen Kanälen wie oft jeweils durch die schweizerische und durch die amerikanische Nutzer-Bevölkerung auf den wichtigsten Kanälen aktiv gesucht werden. Die Einblicke, welche die Studie bietet, ermöglichen den relevanten Einbezug der breiten Bevölkerung in die Gestaltung des digitalen Wandels. Die zuverlässige Wahrnehmung der Chancen und die vertiefte Kenntnis der wichtigsten vorherrschenden Risiken in der Bevölkerung können helfen, eine bessere Einordnung und eine höhere Akzeptanz für neue Technologien allgemein zu erzielen.

Die digitale Transformation ist in vielen Bereichen des Alltags spürbar, und Abläufe verändern sich. Dies führt einerseits zu mehr Effizienz oder gar komplett zu neuen Möglichkeiten, jedoch kann dieser Wandel auch diffuse Ängste oder ernsthafte Risiken mit sich bringen. Die digitale Transformation ermöglicht insbesondere auch sehr viele Chancen: Neue Geschäftsmodelle, zum Beispiel E‑Banking, E‑Commerce oder neue Formen der Nutzung von Telekommunikation, Mobilität, Energie etc. Andererseits drohen Gefahren, welche sich auf die virtuelle Welt auswirken, zum Beispiel im Rahmen von fehlenden Backups, Cyberattacken, oder Hacks und kryptischen Verschlüsselungen, welche neben der Verunmöglichung der weiteren Nutzung, Erpressungsversuchen, oder Datenverlusten, oft nicht einmal auf einen materiellen Schaden abzielen, sondern den Menschen indirekten Schaden zufügen können, wie beispielsweise Cybergrooming (z. B. Online-Kontaktaufnahme durch Erwachsene an Kinder zur Vorbereitung sexuellen Missbrauchs) oder Hate Speech (beleidigende oder drohende Online-Kommunikation zur persönlichen Herabwürdigung, Verunglimpfung bis hin zu Ehrverletzung einzelner Personen oder Personengruppen).

Während Studien oftmals punktuell nur einzelne Aspekte dieses grossen Spektrums untersuchen, oder sich auf bestimmte Kontexte, Einzelereignisse oder Regionen konzentrieren, wird hier dank dem Einsatz von neusten Technologien die Möglichkeit genutzt, einen gesamtheitlichen Ansatz (Scope) zu wählen. Diese wissenschaftliche Methode (siehe Kap. 3) erlaubt es, die Suchanfragen der gesamten Online-Bevölkerung aggregiert zuverlässig zu messen, was im direkten Vergleich 8.016.520 Personen in der Schweiz, und 293.544.605 Personen in den USA entspricht^2^.

Basierend auf diesem Datensatz sollen in dieser Studie die folgenden Forschungsfragen beantwortet werden:

- Welche Chancen und Risiken werden durch die Bevölkerungen in beiden Ländern im selben Zeitraum in Bezug auf digitale Transformation am häufigsten aktiv gesucht?
- Wie unterscheidet sich diese aktive Nachfrage zwischen der Schweiz und den USA?
- Welche unterschiedlichen Kanäle werden am häufigsten verwendet, um Chancen und Risiken zur digitalen Transformation zu suchen?

In diesem Forschungsprojekt wurde der Frage nachgegangen, ob die Nutzer*innen in der Schweiz, gleichbedeutend vermutlich auch in ganz Europa, einer zu starken Risikowahrnehmung von neuen Digitaltechnologien verfallen sind, während selbige in den USA und insbesondere auch in Asien die Chancen der neuen Technologien technisch und kommerziell viel umfassender ausnützen. Unsere Hypothesen gehen von einem starken Bias zumindest in der transatlantischen Wahrnehmung zu diesen *areas of interest* (AOI) aus. Es sollen mit dieser Studie die folgenden Hypothesen validiert werden:

### Hypothese 1:

Seitens der Bevölkerung gemessen an der Internetnutzung besteht eine viel höhere Risikoaffinität bezüglich Cyberthemen in der Schweiz als in den USA

### Hypothese 2:

Chancen der Digitalisierung werden in der Schweiz dadurch viel weniger intensiv genutzt als in den USA

Nachfolgend ist der Artikel wie folgt aufgebaut: Kap. 2 beschreibt den Stand der Forschung auf diesem Gebiet. Danach werden in Kap. 3 die Methode und in Kap. 4 das Vorgehen im Rahmen dieser Studie erläutert. Kap. 5 beschreibt anschliessend die Resultate der Studie, welche in Kap. 6 abschliessend diskutiert werden.

## Stand der Forschung

Die Aspekte der Akzeptanz von digitaler Transformation sind zahlreich. Diverse Studien haben das Thema untersucht, jedoch sind bisherige Studien zu dieser Thematik mit bestimmten Zielgruppen oder einer limitierten Anzahl von Personen durchgeführt worden und ermöglichen daher nur einen vergleichsweise kleinen Einblick in die digitale Transformation als nationale Entwicklung.

Beispielsweise wurde untersucht, wie die digitale Transformation im Bankensektor in Griechenland akzeptiert wird (Kitsios et al. 2021). Diese Studie basiert auf einer Umfrage mit 161 Teilnehmenden aus verschiedenen Stufen und Bereichen, welche Mitglieder der Federation of Banking Employees of Greece (OTOE) sind. Die Ergebnisse der Studie legen nahe, dass ein Grossteil der Teilnehmenden der Meinung ist, dass die Digitalisierung die Arbeit vereinfacht und es ihnen ermöglicht, mehr Arbeit in kürzerer Zeit zu erledigen.

Es wurde auch untersucht, wie Studierende die Gefahren des Internets einschätzen (Van Schaik et al. 2017). Mittels einer quantitativen empirischen Studie im UK und in den USA wurden 16 Sicherheitsgefahren aus dem Internet und das Verhalten 436 Studierender betrachtet. Identitätsdiebstahl, Keylogging (Schadsoftware, welche aufzeichnet, was auf der Tastatur eingegeben wird), Cyber-Bullying (Mobbing, welches online stattfindet) und Social Engineering (zwischenmenschliche Manipulation, zur Herausgabe sensibler Daten wie bspw. Passwörter) wurden zu den wichtigsten Risiken gezählt. 200 Studierende wurden auch in einer anderen Studie aus Deutschland (Bond et al. 2018) untersucht, in Kombination mit 381 Lehrpersonen. Die Wahrnehmung beider Gruppen auf die Digitalisierung und die Verwendung von digitalen Medien wurde hierbei analysiert. Die Studie zeigte, dass, obwohl die digitale Lernplattform als wichtigstes Tool angesehen wurde, weder Studierende noch Lehrpersonen das volle Potenzial der Digitalisierung ausschöpfen.

Während sich andere Auswertungen auf eine bestimmte Zielgruppe fokussieren, und nur eine limitierte Anzahl von Personen untersuchen, erlaubt es der vorliegende Ansatz, eine Vollerhebung über die Schweizer und über die US-Onlinebevölkerung zu generieren. Diese *digital impact KPIs*, welche in dieser Studie angewendet wurden, ermöglichen eine aggregierte längerfristige breit abgestützte Marktforschung in Echtzeit auf rund 200 Suchfeldern pro Land, auf denen sämtliche online aktiven Nutzer*innen systematisch auf benutzte Suchinhalte und auf die benutzten Kanäle untersucht werden, ohne deren Privatsphäre zu tangieren oder sie zu beeinflussen.

## Methode

Die zur Analyse verwendete Software zur Sammlung von Daten basiert auf API^3^- und Suchdaten zur Online-Nutzung von Suchfeldern und ermöglicht eine Unterscheidung zwischen den aggregierten Daten aus verschiedenen Ländern (basierend auf der Domäne). Diese Software wurde über viele Jahre weiterentwickelt und erprobt. Die verwendete wissenschaftlich statistische Mess-Methode ist einzigartig, weil sie keine zusätzlichen Medieninformationen miteinbezieht, sondern direkt die aktiven Suchen der Online-Gemeinschaft misst und validiert, direkt an der Schnittstelle von den relevanten Plattformen der beiden Länder. Aufgrund dieser Unterscheidung ist es ausserdem möglich, festzustellen, auf welchen Kanälen wie häufig bestimmte Schlagworte tatsächlich gesucht wurden.

Die auf diese Weise gesammelten Daten sind besonders wertvoll für die Wissenschaft, aber auch für die Marktforschung, E‑Shops, Produkttrends, Relevanzprüfungen, Werbe- und Website-Wirkungsanalyse, für Digital-Planungen oder für Predictive Analytics (daten-basierte Prognosen) in politischen Kampagnen, sie dienen auch als KPIs für die digitale Wirkungsmessung von gesellschaftlich kollektiv relevanten Themen im Allgemeinen.

Das Suchverhalten der Online-Bevölkerung ändert sich ständig. Schätzungen zufolge nutzen 87,2 % der Schweizer Bevölkerung (ITU 2016) das Internet und damit verschiedene Arten von Suchmaschinen, soziale Medien und E‑Shops. Je nach Zielgruppe und Aktualität wird heute oft nach „Special Interest“-Suchoptionen gesucht, wie z. B. via Snapchat, Facebook, Instagram oder TikTok. Hinzu kommt, dass nach den Erfahrungen der Autor*innen heute durchschnittlich 30–40 % der universellen Suche auf Social Media und ein steigender Prozentsatz auf E‑Shops entfällt. Man berücksichtige dabei, dass beispielsweise Amazon sich in den vergangenen Jahren von einem Buch e‑Shop ebenfalls zu einer Art kommerziellen Suchmaschine entwickelt hat und Google von einer Suchmaschine zu einem e‑Shop, welche zu sämtlichen Inhalten Ergebnisse liefert. Ausserdem kommen ständig neue Suchoptionen hinzu und alte verschwinden. Diese Veränderungen werden für eine differenzierte Online-Forschung deshalb immer wichtiger. Die Leistungsfähigkeit von Suchdaten hat sich in mehreren Anwendungsfällen bewährt, um die Einstellung, die Stimmung oder die Meinung der breiten Bevölkerung zu messen, z. B. um Rassismus während der Wahlen zu untersuchen (Stephens-Davidowitz 2014) oder um Gefühle wie geringes Selbstwertgefühl zu erkennen (Zaman et al. 2019). Es hat sich gezeigt, dass solche validen und zuverlässigen Methoden die Ergebnisse herkömmlicher Umfragen verbessern, da die Teilnehmer*innen bei Umfragen zum Lügen neigen (Stephens-Davidowitz 2017). Ausserdem hat sich gezeigt, dass nur ein bestimmter Teil der Bevölkerung an Umfragen teilnimmt, was zu Verzerrungen der Ergebnisse führen kann (Wright 2005). Im US-Präsidentschaftswahlkampf im Jahr 2016 konnte man mit der Methode der Online-Forschung Mitte September bereits die Wahl von Donald Trump voraussagen, während die NY Times gleichzeitig Umfrageresultate publizierte, welche mit 90 %-tiger Sicherheit eine Wahl von Hillary Clinton zur Präsidentin prognostizierten (Naji et al. 2016).

Die meisten aktuellen Forschungsarbeiten zur Suche beschränken sich auf Google-Daten oder Hashtags in sozialen Medien, mit der starken Einschränkung, dass jeweils nur ein Teil der aktiven Internetbevölkerung erfasst wird. Die hier angewandte Methode verwendet einen neuen kanalübergreifenden Ansatz, bei dem Daten aus 14.103 Quellen weltweit extrahiert werden, darunter Suchmaschinen (Wikipedia, Google, Bing, Yahoo!, Ask, Lycos, Alexa, Technorati, MetaCrawler, Search.com, lokale Suchmaschinen usw.), Plattformen sozialer Netzwerke (z. B. Facebook, Twitter, Linkedin, TikTok usw.) und E‑Shop-Suchen (z. B. Amazon, ebay, Alibaba usw.). Die gesammelten Daten werden systematisch aggregiert und im Quervergleich analysiert, um die von Internetnutzer*innen gestellten Suchanfragen und die Häufigkeit dieser Suchanfragen in den Domänen von 203 Ländern zu ermitteln. Die Möglichkeit, zahlreiche Suchmaschinen und soziale Netzwerke zu untersuchen, zu parametrisieren und zu vergleichen, erlaubt es, valide und verlässliche aggregierte Einblicke in das inhaltliche Suchverhalten der digital aktiven Schweizer und amerikanischen Bevölkerung zu den Cyber Themen zu gewinnen.

In der jüngsten Vergangenheit wurden ähnliche Methoden erneut angewendet, um die Auswirkungen des US-Wahlkampfes 2020 (Glauser et al. 2021) oder beispielsweise die Akzeptanz der Contact Tracing Apps in der Corona Pandemie (Glauser et al. 2020) zu untersuchen.

Die verwendete Methode arbeitet nur mit vollständig anonymisierten Daten, es wurden ausschliesslich öffentlich zugängliche Suchdaten erhoben und keinerlei Nutzer*innen bezogene Daten verwendet. Die Ergebnisse weisen lediglich inhaltsanalytische Daten und Kanal-spezifische Daten nach. Die Daten können als ganzheitlich betrachtet werden, allerdings mit der Einschränkung, dass abhängig von der jeweiligen Domain jeweils ganzheitliche nationale Daten verwendet wurden. Domains mit der Extension *.com sind meistens äquivalent zu den USA Daten bewertet.

**Abb. 1:**
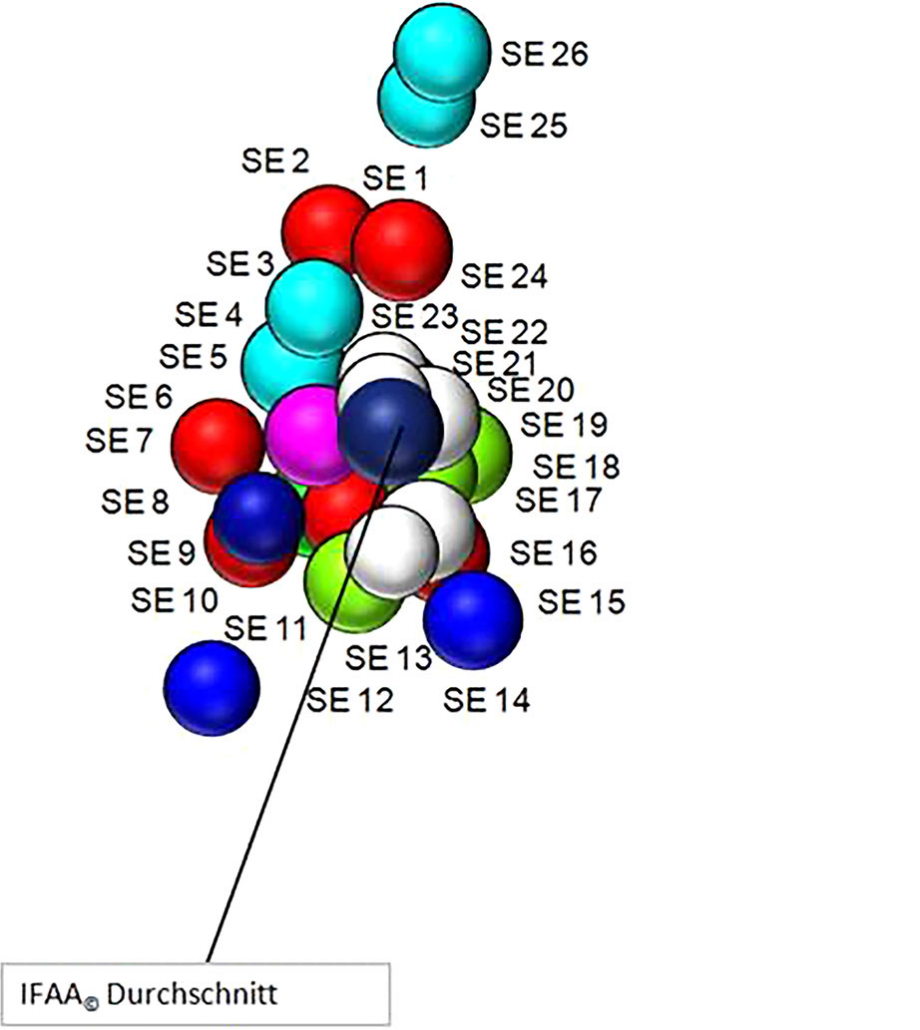
Beispiel der Quervalidierung von Suchmaschinen (search engines SE)

Die Frequenzen der Suchabfragen werden jeweils über alle verfügbaren Kanäle pro Land quer-validiert und im Durchschnitt angegeben. Die Zuverlässigkeit der Daten (*reliability*) wird im Vergleich zu der Anzahl aktiver Internet-Nutzer*innen pro Land gemessen. Während die traditionellen Suchmaschinen jeweils nur eine Komponente messen, erlaubt es die in dieser Studie verwendete Methode, diese als Durchschnittswert anzugeben, wie in Abb. 1 gezeigt.

## Vorgehen

In einem ersten Schritt wurde eine Liste von positiven Schlagworten in Bezug auf Chancen der Cyberwelt, und eine Liste mit negativen Schlagworten zu den entsprechenden Risiken generiert. Die Schlagwörter wurden auf Deutsch (in Hinblick auf die Untersuchung in der Schweiz) und in der Übersetzung in Englisch (für die Untersuchung der USA) erstellt.

Initial wurde die Wortliste basierend auf sog. Wortstammlisten erstellt. Diese Wortstammlisten basieren auf umfassenden *Site Grabbings* (systematische inhaltlich vollständige Erfassung von zahlreichen Webseiten), durch welche umfangreiche nahezu vollständige *Dictionaries* (Wörterbücher von A bis Z der vorhandenen Begriffe) erstellt werden können. Basierend auf diesen Dictionaries und Synonymdatenbanken werden zunächst sämtliche Begriffe rund um „cyber“ in Kombination mit Chancen Referenzierungen oder mit Risikobegriffen in Kombination verwendet. In einem ersten Durchlauf wird der Dictionary vollständig getestet (sämtliche Begriffe (issues) werden auf die derzeitigen Nachfrage hin gemessen), die kollektiv relevanten Wörter (mit Nachfragewerten über einem bestimmten Grenzwert) und die relevanten Kombinationen davon werden für das Projekt längerfristig indiziert und gemessen. Dieses Vorgehen basiert auf dem Ansatz, dass damit die Untersuchung möglichst vollständig und unabhängig ist und näherungsweise sämtliche relevanten Informationen und Kanäle mit einem hohen Referenzwert zu den beiden Themen Cyberchancen und -risiken abdeckt.

Tab. 1 zeigt einige ausgewählte semantische Beispiele auf Deutsch.

**Tab. 1:** Einige ausgewählte Beispiele der deutschen Begriffe

Chancen	Risiken
Dank Online	Digitale Sicherheitslücke
Online Möglichkeiten	Adresse missbrauchen
Zugang für alle	Veröffentlichung von Daten
Digitale Chance	Digitaler Missbrauch
Online Vorteile	Datenschutzgesetz
…	…

Für die Schweiz wurden 143 für Chancen, und 162 Schlagworte für Risiken verwendet, und für die USA 80 für Chancen und 64 Schlagworte für Risiken. Diese Differenz ergibt sich aus der obenstehenden Vorgehensweise, um die kollektiv relevanten Wörter und Begriffe auszuwählen. Für die Auswertung werden jeweils Durchschnittswerte pro Schlagwort oder Kombinationen verwendet, womit die Anzahl der verwendeten Varianten bei den statistischen Vergleichen nicht ins Gewicht fällt.

Es wurde anschliessend untersucht, wie oft und auf welchen Kanälen diese kollektiv relevanten Schlagworte gesucht worden sind, einerseits in der Schweiz, und andererseits in den USA. Der Beobachtungszeitraum erstreckte sich vom 04.08.2021 bis zum 11.10.2021.

Basierend auf den in diesem Kapitel definierten Schlagworten wurde mittels der in Kap. 3 beschriebenen Methode untersucht, wie oft die Schlagworte auf welchen Kanälen gesucht wurden. Die Schlagworte wurden täglich in Bezug auf jeweils die letzten 30 Tage im Durchschnitt („moving averages“) gemessen. Für die Darstellung der Ergebnisse werden stets aggregierte Durchschnittswerte der Messungen verwendet. Es wurden insgesamt 25 Kanäle für die USA und 30 Kanäle für Schweiz untersucht, welches die wichtigsten e‑Shops, sozialen Medien und Suchmaschinen umfasste. Die Anzahl der digitalen Kanäle in der Schweiz ist etwas höher, da auch kleinere lokale Kanäle mitberücksichtigt wurden.

## Resultate

Dieses Kapitel beschreibt die Ergebnisse der oben beschriebenen Studie. Es wird aufgezeigt, wie Chancen und Risiken in der Schweiz und den USA im Suchvolumen sichtbar sind, und es wird ein Vergleich zwischen den beiden Ländern gemacht.

### Chancen und Risiken in der Schweiz

**Abb. 2:**
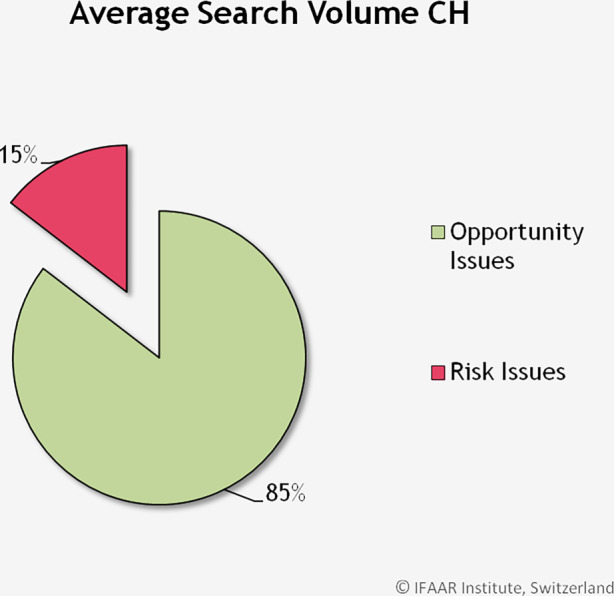
Durchschnittliches Suchvolumen für Cyberrisiken und Cyberchancen in der Schweiz

Im ersten Teil der Studie wird verglichen, inwiefern sich Chancen und Risiken in Bezug auf Cyberthemen in der Schweiz unterscheiden. Abb. 2 zeigt die durchschnittlichen Suchvolumen über den Beobachtungszeitraum, zusammengefasst über alle Schlagwörter für Cyberchancen und Cyberrisiken. Während nur 15 % des Suchvolumens sich auf Risiken bezieht, ist ein Grossteil der Suchanfragen (85 %) für positive Wörter für die Chancen der Digitalisierung.

**Abb. 3:**
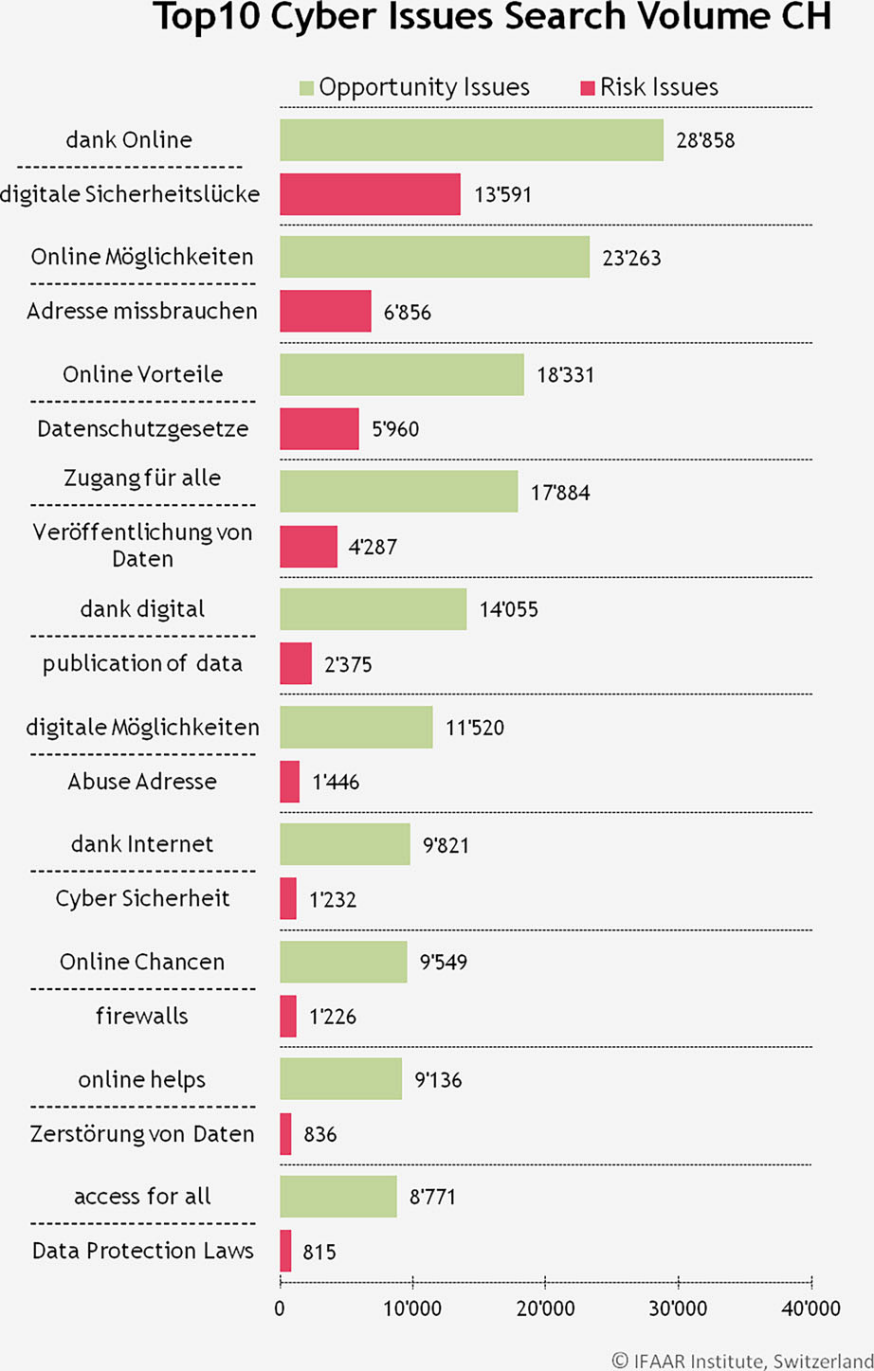
Die 10 Begriffe, welche meistens gesucht wurden in der Schweiz, bezüglich Chancen (opportunity issues) und Risiken (risk issues). Häufig auf Englisch verwendete Begriffe wurden auch für die Schweiz auf Englisch untersucht

Anschliessend wurde untersucht, welches im Detail die meistgesuchten Begriffe sind. Wie in Abb. 3 gezeigt, sind die häufigsten Risiken *digitale Sicherheitslücke, Adresse missbrauchen* und *Datenschutzgesetze*. Als Chancen wurden die Ausdrücke *dank Online, Online Möglichkeiten* und *Online Vorteile* am häufigsten gesucht.

### Chancen und Risiken in den USA

**Abb. 4:**
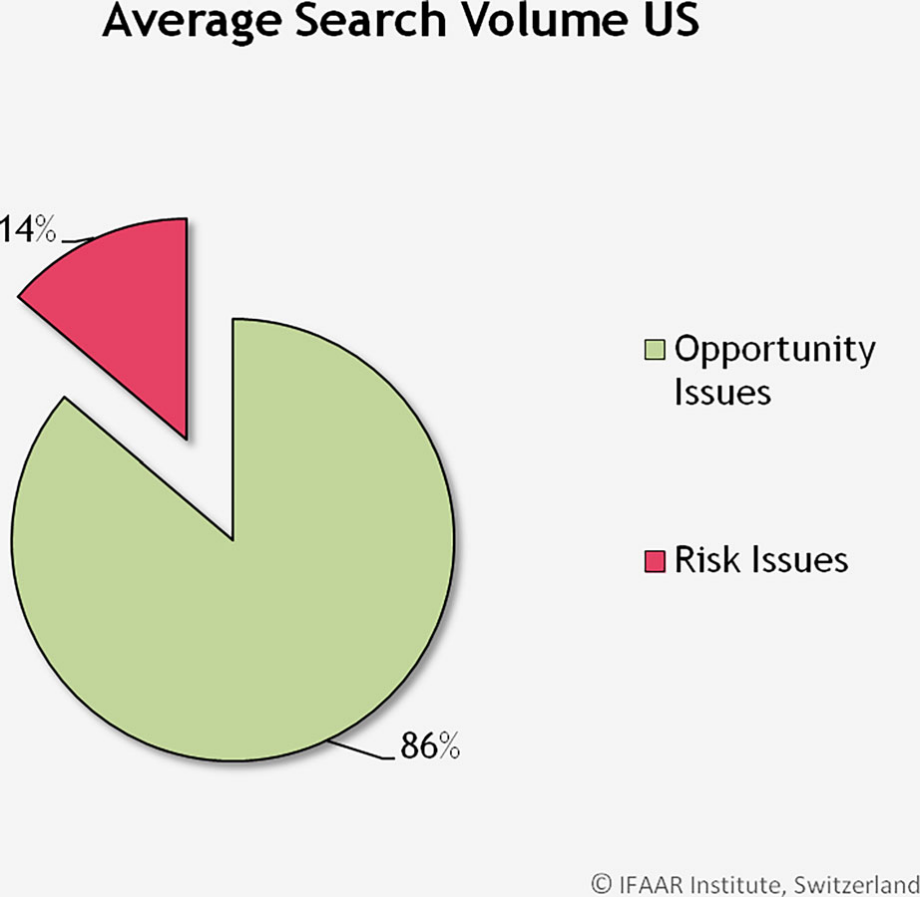
Durchschnittliches Suchvolumen für Cyberrisiken und Cyberchancen in den USA

Im zweiten Teil unserer Studie wird untersucht, inwiefern nach Cyberrisiken und -chancen in den USA gesucht wird. Abb. 4 zeigt die durchschnittlichen Suchvolumen über den Beobachtungszeitraum, zusammengefasst über alle Schlagwörter für Cyberrisiken und Cyberchancen. Während nur 14 % des Suchvolumens sich auf Risiken bezieht, ist ein Grossteil der Suchanfragen (86 %) für positive Wörter, also für die Chancen der Digitalisierung.

**Abb. 5:**
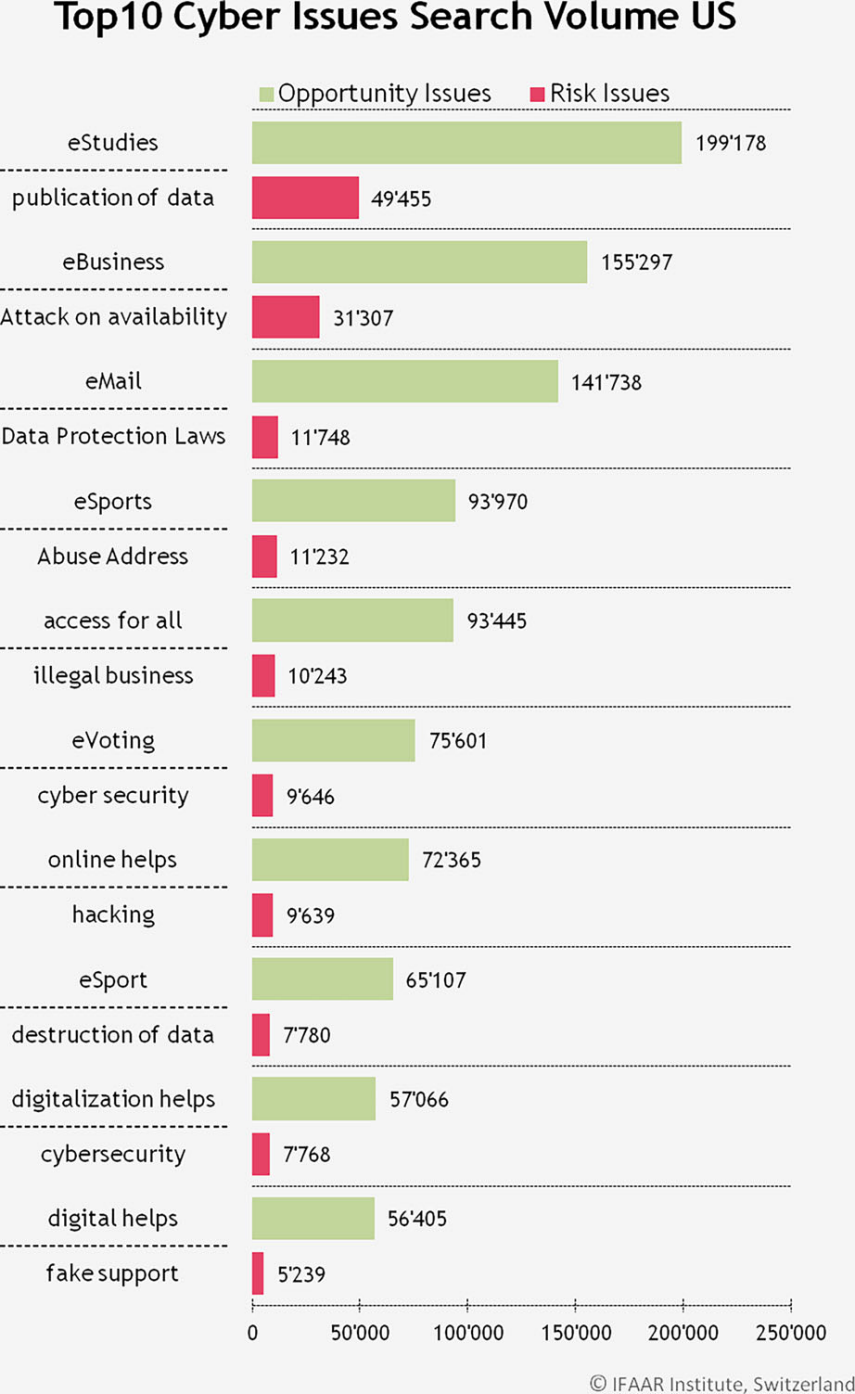
Die 10 Begriffe, welche meistens gesucht wurden in den USA, bezüglich Chancen (opportunity issues) und Risiken (risk issues)

Im nächsten Schritt wird untersucht, welche Risiken und Chancen am häufigsten gesucht wurden, wie in Abb. 5 für die Top-10 dargestellt. Die Wörter *eStudies, eBusiness* und *eMail* haben als Chancen grosse Beliebtheit, während die wichtigsten Risiken *publication of data (dt: Datenveröffentlichung), attack on availability (dt: Angriff auf die Verfügbarkeit)* und *Data Protection Laws (dt: Datenschutzgesetze)* sind. In direktem Vergleich zu den Resultaten aus der Schweiz, sind die gesuchten Chancen im Falle der USA einiges konkreter. Es werden bestimmte Dienstleistungen häufiger gesucht, während in der Schweiz eher allgemeine Formulierungen verwendet werden. Bei den Risiken taucht in beiden Ländern das Datenschutzgesetz unter den Top‑3 Themen auf. Die anderen beiden Topwörter beziehen sich in beiden Fällen auf Cyber-Angriffe, sind aber unterschiedlich ausformuliert.

### Ländervergleich

**Abb. 6:**
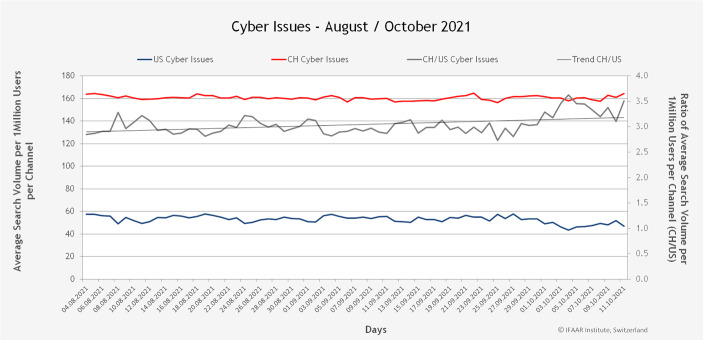
Suchvolumen aller Cyberthemen (Risiken und Chancen) pro 1 Mio. Nutzer*innen

Um eine Vergleichbarkeit der beiden Länder trotz der unterschiedlichen totalen Anzahl an Suchanfragen (aufgrund der unterschiedlichen Nutzer*innenzahl) zu erzielen, werden die Suchanfragen jeweils über die Zeit von 30 Tagen und auf 1 Mio. User pro Kanal normalisiert. Abb. 6 vergleicht das Suchvolumen über den Beobachtungszeitraum für die Schweiz (in rot, oberste Linie) und die USA (in blau, unterste Linie), für alle Cyberwörter (Chancen und Risiken). Jeder Datenpunkt entspricht dem Durchschnittswert aller Wörter bei einer täglichen Messung über die jeweils letzten 30 Tage. Das Verhältnis der Werte für die Schweiz und die USA (CH/US) wird durch die graue Linie mit Skala auf der rechten Seite dargestellt. Dadurch wird aufgezeigt, wie viel höher das Suchvolumen für Cyberthemen in der Schweiz ist.

**Abb. 7:**
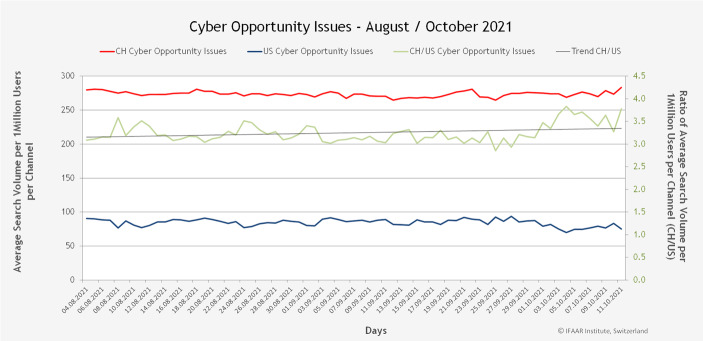
Suchvolumen der Cyberchancen pro 1 Mio. Nutzer*innen

Abb. 7 und 8 zeigen die gleiche Darstellung, aber unterscheiden nach positiven Wörtern (Chancen) und negativen Wörtern (Risiken).

**Abb. 8:**
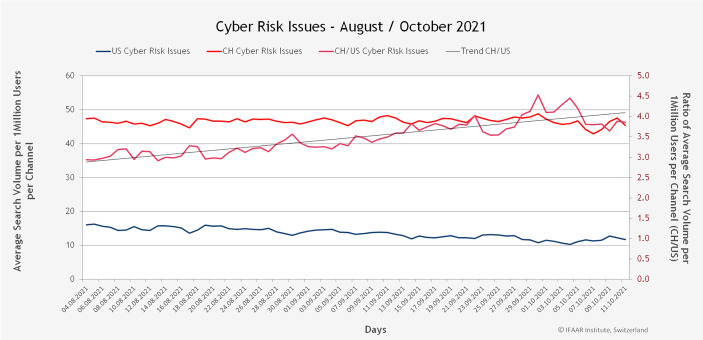
Suchvolumen der Cyberrisiken pro 1 Mio. Nutzer*innen

**Abb. 9:**
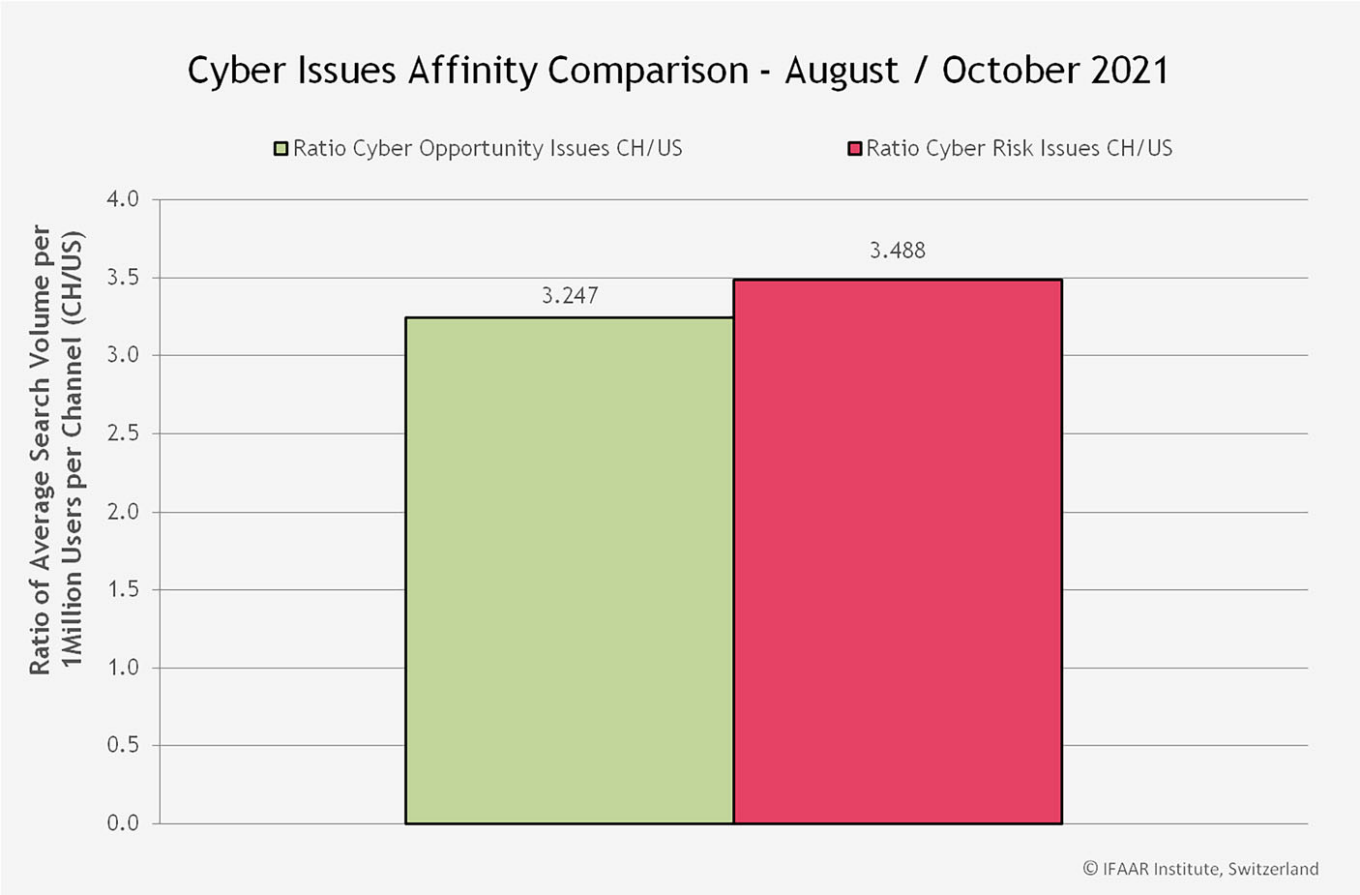
Durchschnittliches Verhältnis zwischen dem Suchvolumen in der Schweiz und den USA

Abb. 9 zeigt das durchschnittliche Verhältnis zwischen dem Suchvolumen in der Schweiz und demjenigen in den USA. In der Schweiz suchen die Nutzer*innen ca. 3,5-mal häufiger nach Risikothemen als in den USA. Etwas weniger stark ist der Unterschied bei den Chancen. Hier suchen Schweizer*innen ca. 3,2-mal häufiger nach Chancen als die Amerikaner*innen. Der Vergleich zeigt auch eine hohe Risikoaffinität der Schweizer Abfragen, es gibt demnach deutlich mehr Suchen nach Risikothemen als in den USA und der mittelfristige Trend geht in der Schweiz nach oben, während die Nachfrage nach den Cyber-Themen in den USA im Trend derzeit sogar leicht rückläufig ist.

**Abb. 10:**
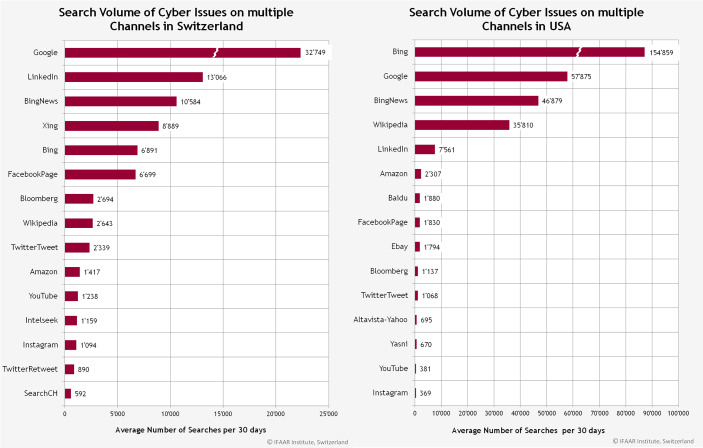
Die am häufigsten verwendeten Kanäle für Suchanfragen in der Schweiz und den USA

Es wurde ausserdem untersucht, welche Kanäle verwendet werden, um die Begriffe zum Thema Digitalisierung zu suchen. Abb. 10 zeigt die Kanäle, welche am häufigsten genutzt werden, um solche Begriffe zu suchen, in der Schweiz verglichen mit den USA.

**Abb. 11:**
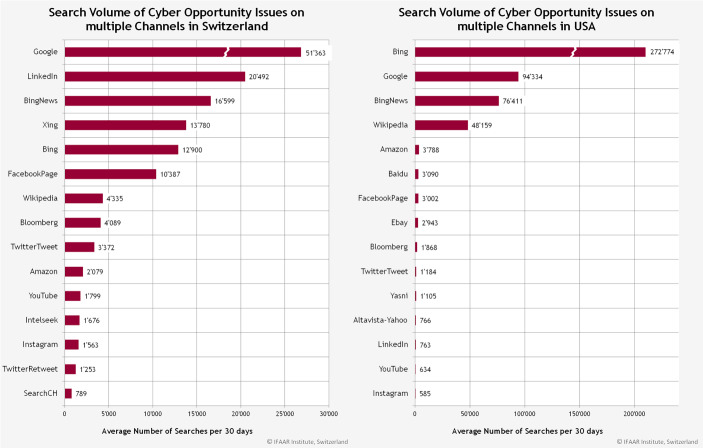
Die am häufigsten verwendeten Kanäle für Suchanfragen für Cyberchancen in der Schweiz und den USA

**Abb. 12:**
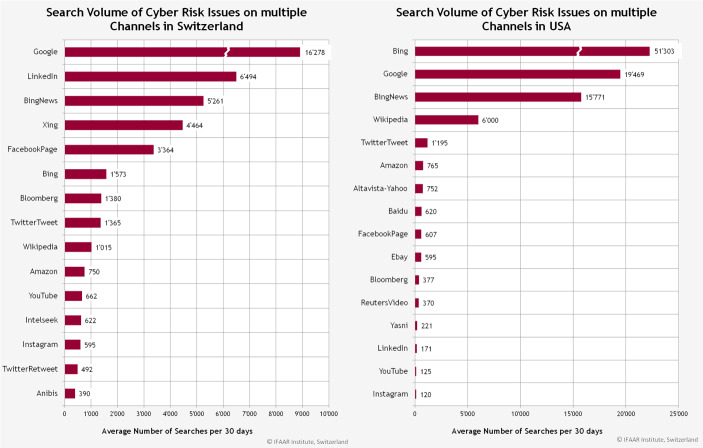
Die am häufigsten verwendeten Kanäle für Suchanfragen für Cyberrisiken in der Schweiz und den USA

In einer separaten Betrachtung lässt sich unterscheiden, wie sich dies für Chancen (siehe Abb. 11) und Risiken (siehe Abb. 12) verhält.

Während in den USA Bing, Google und BingNews in beiden Fällen die Top‑3 Kanäle bilden, wird in der Schweiz Google, Linkedin und BingNews im häufigsten verwendet, um nach spezifischen Cyberbegriffen zu suchen.

## Diskussion

Es konnte in unserer Studie nachgewiesen werden, dass im Verhältnis zur Anzahl Personen (normalisiert auf 1 Mio. Nutzer*innen), Begriffe oder Kombinationen von Begriffen in Bezug auf Digitalisierung und Cyberthemen in der Schweiz insgesamt öfter gesucht werden als in den USA. Insbesondere ist dies bei den Risiken der Fall: dort wurde in der Schweiz signifikant, insgesamt knapp 3,5-mal häufiger, nach entsprechenden Begriffen gesucht. Dieses Ergebnis unterstützt die Hypothese 1, welche davon ausging, dass Risiken bezüglich der Digitalisierung in der Schweiz unter den Nutzer*innen viel stärker ausgeprägt sind. Hypothese 2, welche davon ausgeht, dass auch die Chancen der Digitalisierung in den USA wesentlich häufiger nachgefragt und genutzt werden, konnte durch unsere Studie indessen nicht bestätigt werden. Die digitale Nachfrage nach den Cyberchancen ist in der Schweiz sogar höher als in den USA.

Worauf ist dieser Unterschied zwischen den beiden Ländern zurückzuführen? Ist es ein kultureller Unterschied, welcher es in den USA erlaubt, weniger risikoaffin bezüglich der Cyberthemen zu sein? Sind die Amerikaner*innen tatsächlich mutiger, wenn es darum geht für die Nutzung von Chancen allenfalls gewisse Risiken in Kauf zu nehmen? Dies muss durch weitergehende wissenschaftliche Analysen untersucht werden. Insbesondere kann dies erreicht werden, indem die vorliegende Studie auf weitere Länder ausgedehnt würde. Insbesondere die EU Länder oder der asiatische Raum könnten hier zusätzlich in die Analyse miteinbezogen werden. Unser Vergleich, der Schweiz mit den USA, ermöglicht einen ersten Einblick, und zeigt auf, wie vielversprechend solche neuen Methoden und digitale Wirkungs-KPIs eingesetzt werden.

In der Vergangenheit wurde oftmals der Begriff *digital divide* verwendet, wenn auch nicht immer einheitlich (Scheerder et al. 2017). Bei dem *digital divide* wird primär davon ausgegangen, ob ein Internetanschluss verfügbar ist und entsprechend genutzt wird, respektive inwiefern dieser Zugang einer Gruppe von Menschen verwehrt bleibt. Aufgrund der massenhaften Verbreitung von Smartphones mit Internetanschluss und des dadurch sehr hohen Bevölkerungsanteils in der Schweiz, welcher regelmässig das Internet nutzt (über 93 % gemäss ITU 2016), ist dies nicht mehr wirklich ein entscheidender Faktor in der Schweiz. Es gilt trotzdem zu bemerken, dass im Rahmen der Covid-19 Krise noch *digital-divide*-Problematiken festgestellt werden konnten (z. B. Seifert et al. 2020), insbesondere auch in Bezug auf ältere Personen ohne Internetzugang (Seifert 2020) oder in Bezug auf den Fernunterricht bei den Schulen (Sosa Díaz 2021). Die vorliegende Studie hat gezeigt, dass die Cyber-Risiken als sehr relevant wahrgenommen werden, und ein entsprechend hohes Suchvolumen generieren. Es handelt sich daher nicht um ein *digital divide* im Sinne von *können oder Zugang haben* (d. h. die entsprechenden Ressourcen wie Internet oder Hardware zu Verfügung zu haben), sondern eher von einem *wollen*. Die Autor*innen der Studie beobachten ein bewusstes kritisches Hinterfragen der neuen Technologien durch viele Nutzer*innen und eine intensive positive Nutzung durch die andere Nutzer*innengruppe, welches sie daher neu *cyber divide* nennen. Sie gehen davon aus, dass ihre Resultate verschiedene gesellschaftliche Gruppen abbilden, mit einer Affinität zum Thema Digitalisierung, einerseits zu den Risiken und andererseits zu den Chancen von ICT.

Insbesondere gilt es nun zu untersuchen, was die Auswirkungen von dieser Risikoaffinität sind. Weitergehende Studien sollten daher untersuchen, inwiefern diese Konzentration auf Risiken sich auf die Wettbewerbsfähigkeit der Schweiz in Bezug auf Digitalisierung auswirkt, und ob allenfalls Chancen nicht im gleichen Ausmass genutzt werden, wie dies in anderen Ländern der Fall ist. Zur Auswertung solcher Risiken und Chancen sind objektive, daten-basierte Methoden unabdingbar, wie sie in dieser Studie verwendet wurden. Eine subjektive Debatte basierend auf Einzelmeinungen oder durch die Medien punktuell stark hochstilisierten Einzelereignissen, kann bei einem solchen Thema mit hohem emotionalem Anteil problematisch sein und die öffentliche Meinung bestenfalls nur einseitig beeinflussen.

Diese Studie hat das Potenzial solcher digitalen KPIs im Vergleich zu traditionelleren Instrumenten wie Umfragen gezeigt. Statt Einzelmeinungen können grosse Teile der Bevölkerung einbezogen werden. Das Konzept eines *cyber divide*, welches auf den Ergebnissen dieser Studie definiert wurde, gibt einen ersten interessanten Hinweis auf eine Gruppensegmentierung der digitalen Gesellschaft, welche in der künftigen Forschung weitergehend untersucht werden sollte. Dies suggeriert ausserdem rechtliche sowie psychologische Fragestellungen in Hinblick auf die Relevanz der Risikoeinschätzung, und wie sich diese beiden Gruppen unterscheiden.

## Footnotes

1 KPIs für key performance indicators.

2 Die Zahlen setzen sich aus Einwohnerzahl und prozentuale Internet User zusammen: ITU https://www.itu.int/en/ITU-D/Statistics/Documents/statistics/2021/July/PercentIndividualsUsingInternet.xlsx, US Census Bureau https://www.census.gov/quickfacts/fact/table/US/PST045219, BFS https://www.bfs.admin.ch/bfsstatic/dam/assets/13707204/master, Stand 2019.

3 API für application program interface, deut. Schnittstelle.
